# A case study of a real-time evaluation of the risk of disease transmission associated with a failure to follow recommended sterilization procedures

**DOI:** 10.1186/2047-2994-3-4

**Published:** 2014-01-21

**Authors:** Curtis J Donskey, Marian Yowler, Yngve Falck-Ytter, Sirisha Kundrapu, Robert A Salata, William A Rutala

**Affiliations:** 1 Geriatric Research, Education, and Clinical Center, Cleveland VA Medical Center, Cleveland, Ohio, USA; 2 Case Western Reserve University School of Medicine, Cleveland, Ohio, USA; 3 Sterile Processing Service, Cleveland VA Medical Center, Cleveland, Ohio, USA; 4 Gastroenterology Section, Cleveland VA Medical Center, Cleveland, Ohio, USA; 5 Department of Medicine, University Hospitals Case Medical Center, Cleveland, Ohio, USA; 6 Department of Hospital Epidemiology, University of North Carolina Health Care, Chapel Hill, North Carolina, USA

**Keywords:** Sterilization, Surgery, Contamination, Failure, Risk analysis

## Abstract

**Background:**

Failures to follow recommendations for reprocessing of surgical instruments may place patients at risk for exposure to pathogenic microorganisms. When such failures occur, medical facilities often face considerable uncertainty and challenges in assessing the actual risks of disease transmission.

**Methods:**

In 2011, staff at an Ohio hospital determined that surgical instruments inside a Steriset Container had inadvertently been autoclaved on a gravity cycle rather than on the recommended pre-vacuum cycle, potentially exposing 72 patients who underwent surgery with the instruments to risk of infection. To provide an assessment of the level of risk, we tested the effectiveness of the machine washer/disinfector step and of the sterilization process inside the Steriset Container on the gravity cycle for killing of *Geobacillus stearothermophilus* spores, *Clostridium difficile* spores, and methicillin-resistant *Staphylococcus aureus* (MRSA). Based on the test results, the risk of transmission of MRSA by the instruments was calculated and the risk of transmission of hepatitis B virus was estimated.

**Results:**

The machine washer/disinfector consistently reduced MRSA recovery by a factor of 1:100,000. The sterilization process inside the Steriset Container consistently reduced MRSA concentrations by a factor of >1:10,000,000 and killed 10^5^*C. difficile* spores and 10^5^*G. stearothermophilus* spores. The risk of MRSA transmission due to the incident was calculated to be 1 in 100 trillion.

**Conclusions:**

The risk for transmission of infection due to the failure to follow recommended sterilization processes was negligible based upon complete killing of *G. stearothermophilus* biological indicator spores, *C. difficile* spores, and MRSA under conditions that replicated the incident where proper procedures were not followed. Such real-time assessments of the risks associated with specific incidents may provide evidence-based information that can be used to inform decisions regarding disclosure of the incident to patients.

## Background

In the United States, approximately 46.5 million surgical procedures and even more nonsurgical invasive medical procedures are performed each year [1,2]. Because these procedures involve contact with sterile tissues or mucous membranes of patients, it is critical that re-usable surgical instruments or devices be appropriately sterilized or disinfected between procedures [1-3]. Failure of staff to follow proper disinfection and sterilization processes are not uncommon, and the number of reprocessing failures that are published or reported in the press represent only a small proportion of the incidents that result in patient notification [1]. Moreover, patients may not be notified in many incidents of failure to follow reprocessing recommendations because the risk of transmission of infection may be considered negligible [1]. When failures occur, medical facilities often face considerable uncertainty in assessing the actual risks of disease transmission associated with a particular deviation from standard recommendations. Unfortunately, real-time assessments of the risks associated with specific incidents are usually not available in time to inform decisions on whether disclosure is indicated or, if disclosure is made, to provide patients with evidence-based data on the risk that they might acquire an infection.

In 2011, Sterile Processing Department staff at an Ohio hospital identified two Steriset Sterilization Containers (Wagner GmbH, Munchen, Germany) containing reprocessed surgical instruments in which a steam chemical integrator strip (Comply Sterigage Steam Chemical Integrator, 3M, St. Paul, MN) indicated that sterilization had not been achieved. Upon review, it was determined that for two autoclave runs on the day in which the Sterisets had been processed, the autoclave had been run on a gravity cycle rather than on the pre-vacuum cycle recommended for Steriset Containers by the manufacturer. Because operating room staff did not routinely document the results of integrator strips placed inside each Steriset Container, it was calculated that up to 72 patients may have undergone operations using instruments that had been autoclaved on the gravity cycle. In the absence of data on how the Steriset Container might function during a gravity cycle, there was uncertainty regarding whether sterilization could be achieved, including questions of whether any steam could enter the container on the gravity cycle and whether pockets of “cold” air might be present within the container. Three of us (C.J.D., R.A.S., and W.A.R.) were consulted for an opinion on the exposure risk associated with this incident. To provide timely information for hospital administrators, infection control personnel, and exposed patients, we performed an investigation to test the hypothesis that sterilization may be achieved inside the Steriset Container on a gravity cycle.

## Methods

### Study setting

The hospital performs a wide range of surgical procedures. All surgical instruments at the facility are reprocessed in a centralized Sterile Processing Department. The first step in reprocessing includes cleaning and disinfection in a machine washer/disinfector (Reliance 444 or Reliance Synergy Washer/Disinfector, STERIS Corporation, Mentor, Ohio). During this step, instruments pass through 5 chambers, including 1) pre-washing with water and enzymatic solution for 1 minute, 2) washing using detergent solution at 150°F for 4 minutes, 3) ultrasonic cleaning with detergent for 4 minutes, 4) thermal and lubricant rinse with water at 180°F for 1 minute followed by instrument mild lubricant at 180°F, and 5) drying for 4 minutes at 180°F. The final step in reprocessing is steam sterilization. For instruments sterilized inside Steriset Containers, the standard operating procedure for the facility indicates that the autoclave (Amsco Eagle, STERIS Corporation) should be set on the pre-vacuum cycle as recommended by the manufacturer.

### Evaluation of the effectiveness of the washer/disinfector

The evaluation was not conducted as a research project and no human subjects were involved. The Cleveland VA Medical Center’s Research and Development Committee reviewed the manuscript and approved submission for publication. We evaluated the effectiveness of the washer/disinfector for removal of vegetative bacteria and spores from surgical instruments. The instruments used for this evaluation were identical to those that were in the Steriset Containers when the failure to follow recommended procedures occurred; all of the instruments were non-hollow and without channels (e.g., retractors, clamps, forceps, needle holders, and scissors). For assessment of removal of vegetative bacteria, the ends of surgical instruments (e.g., retractors, clamps) that contact patients’ tissues were suspended for 1 minute in overnight cultures of clinical isolates of methicillin-resistant *Staphylococcus aureus* (MRSA) (pulsed-field gel electrophoresis type USA 300) or vancomycin-resistant *Enterococcus* (VRE) (C68, a VanB-type VRE isolate) containing 9 log_10_ colony-forming units (CFU) of bacteria per mL. For assessment of removal of spores, the instruments were suspended for 1 minute in suspensions containing 10^6^ CFU per milliliter of non-toxigenic *Clostridium difficile* (American type culture collection #43593) spores. The contaminated instruments were processed in the washer/disinfector as recommended by the manufacturer; control instruments were contaminated in an identical fashion but were not processed in the washer/disinfector.

Survival of *C. difficile* spores was assessed using broth enrichment cultures in *C. difficile* brucella broth (CDBB) [4] and survival of VRE and MRSA was assessed using broth enrichment cultures in brain-heart infusion broth. The inoculated instruments were suspended for 1 minute in the nutrient broth with vortexing and growth was assessed after 48 hours of incubation by plating onto *C. difficile* brucella agar (CDBA) [4] for *C. difficile* or 5% sheep blood agar plates for MRSA and VRE. The limit of detection for the organisms was ~1 log_10_CFU per mL, as determined by assessing recovery of serially diluted preparations of the test organisms applied to instruments.

### Evaluation of the effectiveness of sterilization inside the Steriset Container on the gravity cycle

We evaluated the effectiveness of sterilization inside the Steriset Container when run on the gravity cycle. The autoclave settings were identical to those for the two autoclave runs that had occurred in the incident when the integrators indicated inadequate sterilization (i.e., 270°F for 15 minutes, pressure 28 to 30 psig). Temperature and pressure monitoring equipment are included features of the Amsco autoclave and records of temperature and pressure are maintained for each run of the autoclave; review of the monitoring strips from the time of the incident confirmed that a temperature of 270°F had been achieved for 15 minutes at a pressure of 28 to 30 psig. In addition, the number of instruments and loading pattern inside the container was the same as for the incident (i.e., the Steriset Containers ranged from half full to completely full with non-hollow instruments including forceps, needle holders, scissors, retractors, clamps). The loading pattern of the autoclave for the test runs was designed to replicate the loading pattern during the incident (i.e., fully loaded autoclaves with 9 to 12 Steriset Containers per load). In 4 of the 5 experimental runs included in the analysis, an Attest Rapid Readout Steam Pack (3M, Saint Paul, MN) containing 10^5^*Geobacillus stearothermophilus* spores wrapped in paper was included inside the autoclave but outside the Steriset Containers.

Four methods were used to assess sterilization inside the Steriset Containers. These included: 1) 3M Comply (SteriGage) Steam Chemical Integrators, 2) Biological indicator (BI): 3M Attest1292 Rapid Readout BI/Steam containing *Geobacillus stearothermophilus* spores, 3) *C. difficile* spores (10^5^ CFU in 0.1 mL of sterile water), and 4) MRSA (9 log_10_CFU in 1 mL of brain-heart infusion broth). In addition to the suspensions of MRSA and *C. difficile* in capped glass tubes, multiple instruments were suspended for 1 minute in bacterial suspensions of MRSA and *C. difficile* spores as described previously and placed into Steriset Containers. Survival of *C. difficile* spores or MRSA was assessed using broth enrichment cultures in *C. difficile* brucella broth (CDBB) [4] or brain-heart infusion broth, respectively (i.e., sterile nutrient broth was added directly to the tubes that contained the organisms or the inoculated instruments were suspended for 1 minute in the nutrient broth with vortexing and growth was assessed after 48 hours of incubation by plating onto *C. difficile* brucella agar (CDBA) [4] for *C. difficile* or 5% sheep blood agar plates for MRSA).

To address the question that was raised regarding the possibility that there might be “cold pockets” of air inside the Steriset Container, we used thermal probes (Max Temperature Tester, American Dental Accessories (Minneapolis, MN), to compare the temperature inside versus outside the container in the autoclave and placed biological indicators (i.e., 3M Attest 1292 Rapid Readout BI/Steam containing *G. stearothermophilus* spores) in multiple locations within the Steriset Container (i.e., each corner of the container and in the middle). Acceptance of the steam chemical integrator placed inside the Steriset Container was interpreted as an indication that some steam had entered the container (i.e., the pellet in the integrator has a melting point of 285˚F and will only melt at the 270˚F operating temperature of the autoclave if some steam has entered because steam lowers the melting point).

### Calculation of the risk of transmission of MRSA and hepatitis B virus

We used the method of Rutala and Weber [1] to calculate the risk of transmission of MRSA by the surgical instruments that were inadvertently autoclaved on the gravity cycle. The reductions in MRSA for the washer/disinfector (1:1,000,000) and for the gravity autoclave cycle inside a Steriset Container (1:10,000,000) were based on the data presented here. The likelihood of MRSA contamination of the instruments prior to reprocessing was based on a 15% prevalence of MRSA carriage among inpatients at the hospital. MRSA was chosen because it is a common cause of surgical site infections and the usual perioperative prophylaxis regimen used in the hospital (i.e., cefazolin) would not be protective against this organism. We also estimated the risk of transmission of hepatitis B virus using the same methods.

### Statistical analysis

Fisher’s exact test was used to compare categorical data.

## Results

### Effectiveness of the washer/disinfector

The machine washer/disinfector consistently reduced levels of MRSA and VRE on surgical instruments (N = 15 for each pathogen) to below the limit of detection (<1 log_10_CFU), whereas control contaminated instruments were all positive for contamination with the inoculated pathogens (*P* < 0.0001). *C. difficile* spores were detected on 5 of 15 (33%) of contaminated instruments that were subjected to the washer/disinfector, versus 15 of 15 (100%) control contaminated instruments (*P* = 0.0002).

### Effectiveness of sterilization inside the Steriset Container on the gravity cycle

Table 1 provides a summary of the results of tests of the effectiveness of sterilization on the gravity cycle. Of the 5 test runs, 4 had fully loaded autoclaves with 9 to 12 Steriset Containers per load as was the case in the incident where the failure to follow recommended procedures occurred. Overall, only 4 of 42 (10%) of the integrators inside the containers were accepted, suggesting that insufficient steam entered those Steriset Containers to both melt the pellet in the integrator and cause the chemical to reach the acceptance line. For several of the integrators, there was evidence that the pellet had melted but that heat and steam conditions were not sufficient to cause melting such that the integrator would be accepted. Despite the lack of acceptance of the steam chemical integrator in a majority of containers, sterilization was achieved in 100% of the containers based on killing of the *G. stearothermophilus* biological indicator spores. Moreover, cultures of *C. difficile* spores and MRSA were negative in 23 of 23 Steriset Containers after autoclaving, whereas the positive controls outside the autoclave were all positive for *C. difficile* spores or MRSA. For 1 autoclave run, *C. difficile* spores and MRSA were placed both in the center of the container and in 1 corner of the container; all organisms were killed in both locations. Finally, the maximal temperatures measured by thermal probes placed in multiple locations inside (center of load and at the corners of the container) and outside the Steriset Containers during a gravity cycle were equivalent (270–274˚F).

**Table 1:** Effectiveness of sterilization inside the Steriset Container on the gravity cycle

**Autoclave run**	**Full autoclave load (# Steriset Containers)**	**Integrator accepted, no. (%)**	**Biological indicator ( *Geobacillus stearothermophilus *) killed, no. (%)**	***Clostridium difficile *** **spores killed, no. (%)**	**Methicillin-resistant *Staphylococcus aureus *** **killed, no. (%)**
1	No (1)	1 (100)	1 (100)	1 (100)	1 (100)
2	Yes (12)	1 (8)	12 (100)	3 (100)	3 (100)
3	Yes (10)	0 (0)	10 (100)	10 (100)*	10 (100)*
4	Yes (10)	0 (0)**	10 (100)	6 (100)	6 (100)
5	Yes (9)	0 (0)**	9 (100)	3 (100)	3 (100)
Total		2/42 (5)	42/42 (100)	23/23 (100)	23/23 (100)

*For this run, *C. difficile* spores and MRSA were placed both in the center of the Steriset Container and in corners of the containers with complete killing of the organisms in all locations.

**Several of the integrators had black lines present that indicated melting of the steam chemical integrator pellet, but heat and steam conditions were not sufficient to cause melting such that the integrator would be accepted.

### Calculation of the risk of transmission of MRSA

Table 2 shows the calculated risk of transmission of MRSA by surgical instruments inadvertently autoclaved on the gravity cycle. In addition to the efficacy of the washer/disinfector and the sterilizer, factors such as desiccation may contribute to reductions in numbers of vegetative bacteria on surfaces [5], and authors’ unpublished data. The estimated risk of transmission of blood-borne viruses would be lower than the risk of MRSA transmission based on the prevalence of such viruses in the U.S. population (i.e., hepatitis B s antigen prevalence, 0.5%; hepatitis C, 1.6%; and HIV, 0.37%) [1,6-8]. In addition, these enveloped viruses are in general more susceptible to disinfectants and heat than vegetative bacteria [2,9].

**Table 2:** **Risk assessment for transmission of methicillin-resistant *Staphylococcus aureus *(MRSA) (*A*) and hepatitis B (*B*) by instruments autoclaved inside the Steriset Container on a gravity cycle**

*(A)* MRSA		
**Factors contributing to exposure risk**	**Calculated risk**	**Supporting reference or data**
MRSA prevalence in hospital inpatient population (15%)	15:100	Infection Control Department data from the involved hospital
Risk of transmission via contaminated instruments	1:1	Assume highest risk
Likelihood that contaminated instrument was used	1:1	Assume highest risk
Efficacy of washer/disinfector	1:100,000	Table 1. Consistent removal of 99.999% or more of inoculated MRSA
Effect of MRSA desiccation	1:10	[5] and authors’ unpublished data
Effect of autoclaving inside the Steriset Container on gravity cycle (270 F for 15 minutes, pressure 28–30 psig)	1:10,000,000	Table 1. Consistent killing of 10^8^ colony-forming units (CFU) of MRSA in liquid suspension (limit of detection <1 log_10_CFU/mL)
Individual risk	~1 × 10^-14^ (1 in 100 trillion)	
*(B)* Hepatitis B virus (HBV)*		
**Factors contributing to exposure risk**	**Calculated risk**	**Supporting reference or data**
Prevalence of hepatitis B (HBsAg positive) in U.S. population (0.5%)	5:1,000	[1,5,6]
Risk of transmission via contaminated instruments	1:1	Assume highest risk
Likelihood that contaminated instrument was used	1:1	Assume highest risk
Efficacy of washer/disinfector (removes 99.999% of vegetative bacteria)**	1:100,000	[1] and Table 1
Effect of HBV desiccation	1:1	[1]
Effect of autoclaving inside the Steriset Container on gravity cycle (270 F for 15 minutes, pressure 28–30 psig)	1:10,000,000	Table 1**
Individual risk	~1 × 10^-14^ (1 in 100 trillion)	

*The risk of transmission of hepatitis C or HIV would be lower than the risk of transmission of hepatitis B.

**Enveloped viruses have greater susceptibility to disinfectants and heat than vegetative bacteria such as methicillin-resistant *Staphylococcus aureus* and vancomycin-resistant enterococci.

## Discussion

Although failure to follow recommended sterilization practices is not uncommon, documented transmission of pathogens due to these incidents is very rare [10,11]. Nevertheless, such failures result in considerable uncertainty for infection control practitioners and hospital administrators striving to balance the need to ensure patient safety and the desire to prevent unnecessary anxiety for exposed patients whose risks may be negligible. In the incident investigated here, there was uncertainty regarding whether sterilization might have been achieved despite the failure to autoclave the instrument containers on the correct cycle. Here, we demonstrated experimentally that the risk associated with this failure to follow recommended sterilization practices was essentially zero. Although the hospital involved initially planned to disclose the incident to exposed patients, the experimental data presented here resulted in a reversal of this decision.

The negligible risk associated with this incident is in large part attributable to the fact that modern sterilization processes such as steam sterilization have an enormous margin of safety [2,3]. Previous studies have demonstrated that the levels of microbial contamination on surgical instruments after standard machine cleaning is very low, with 72% of instruments having 0 to 10 colony-forming units of relatively nonpathogenic bacteria (i.e., coagulase-negative *S. aureus*, *Bacillus* spp., and diphtheroids) [12]. We demonstrated that the machine washer/disinfector step used prior to sterilization is extremely effective in eliminating a large burden of vegetative bacteria. Even in the absence of steam entry into the Steriset Container, heating to 180˚F in the washer/disinfector and to 270˚F for 15 minutes in the sterilizer would have been very effective in killing vegetative bacteria and viruses [2,7]. Moreover, our results clearly demonstrate that sterilization based on killing of *G. stearothermophilus* spores and *C. difficile* spores was consistently achieved inside the Steriset Container on the gravity cycle despite only a minority of steam chemical integrator results indicated adequate sterilization.

One important aspect of our investigation is that the primary findings were available within days of the discovery of the incident and were available to inform the decision regarding disclosure of the incident to patients and/or to provide patients with an evidence-based assessment of their level of risk. Previous simulations of failures to follow recommended disinfection and sterilization procedures have provided valuable information on the level of risk, but the findings have usually not been available in a timely manner [13]. Infection Control programs should consider such rapid evaluations when limited data are available regarding the risks associated with specific failures to follow recommended sterilization or disinfection procedures. These evaluations may be simple to conduct given the availability of validated biological indicators that are routinely used in sterile processing departments. The ability to replicate the same disinfection or sterilization conditions using the same equipment that was involved in the incident is an important advantage of such on-site assessments.

Our study has some limitations. First, although our assessment demonstrates that sterilization can be achieved inside the Steriset Container on the gravity autoclave cycle, we cannot state with complete assurance that sterilization was achieved for the runs in question since no biological indicator was included. Second, although we demonstrated that the risk of transmission of vegetative bacteria and viruses is essentially zero, we cannot state with complete assurance that spores were killed. Notably, the only previous report of an outbreak of infections after failure to follow recommended sterilization practices in the United States was an outbreak of *C. perfringens* surgical wound infections [9]. However, spore-forming bacteria are a very rare cause of postoperative wound infections or postoperative sepsis. In the current incident, the hospital involved in the incident reported that none of the exposed patients developed infections with spore-forming organisms during 30 days of follow-up after surgery.

## Conclusion

An experimental investigation confirmed the hypothesis that sterilization may be achieved inside the Steriset Container on a gravity cycle, thus demonstrating that the risk for transmission of infection due to a failure to follow recommended sterilization processes was negligible. Such real-time assessments of the risks associated with specific incidents may provide evidence-based information that can be used to inform decisions regarding disclosure of the incident to patients.

## Competing interests

CJD is a consultant for Merck, 3M, and GOJO and has received research grants from ViroPharma, Ortho-McNeil, Pfizer, and STERIS. WAR is a consultant for Advanced Sterilization Products and Clorox. RAS has received research support from STERIS.

## Authors’ contributions

CJD, MY, Y F-Y, RAS, and WAR made substantial contributions to conception and design, MY and SK made a substantial contribution to acquisition, analysis and interpretation of data. CJD drafted the manuscript, and all authors gave final approval of the version to be published.
